# Time-course based assessment of patient factors and their relationship to chemotherapy induced peripheral neuropathy

**DOI:** 10.3389/fpain.2026.1619858

**Published:** 2026-01-30

**Authors:** Carla Bou Dargham, Ken B. Johnson, Alper Sen, Bihua Bie, Emily E. Rhoades, Jacob Steenblik, Courtney Hershberger, Mei Wei, N. Lynn Henry, Anukriti Sharma, G. Thomas Budd, Joseph Foss, Daniel M. Rotroff

**Affiliations:** 1 Department of Quantitative Health Sciences, Cleveland Clinic Research, Cleveland Clinic, Cleveland, OH, United States; 2 Department of Anesthesiology, University of Utah, Salt Lake City, UT, United States; 3 Department of Anesthesiology, Cleveland Clinic, Cleveland, OH, United States; 4 Taussig Cancer Institute, Cleveland Clinic, Cleveland, OH, United States; 5 Huntsman Cancer Institute, University of Utah, Salt Lake City, UT, United States; 6 University of Michigan Medical School, Ann Arbor, MI, United States; 7 Cleveland Clinic Lerner College of Medicine, Case Western Reserve University, Cleveland, OH, United States; 8 Case Comprehensive Cancer Center, Case Western Reserve University School of Medicine, Cleveland, OH, United States; 9 Center for Quantitative Metabolic Research, Cleveland Clinic, Cleveland, OH, United States

**Keywords:** anxiety, cancer, chemotherapy, depression, neuropathy, patient reported clinical outcomes, physical functioning

## Abstract

**Background:**

Patients commonly experience chemotherapy-induced peripheral neuropathy (CIPN) as an adverse effect from chemotherapies, such as taxanes. In some patients, CIPN symptoms are severe and significantly impact their quality of life. Gaining a deeper understanding of how patient factors change over time, and identifying predisposing patient factors for CIPN susceptibility, could provide opportunities to mitigate the risk of CIPN and improve patient outcomes.

**Methods:**

A total of 229 patients with breast cancer receiving taxane chemotherapy completed study visits over 12 months. Data from patient reported outcomes (PROs) were collected from validated questionnaires to assess CIPN, anxiety, depression, weight, physical function, and sleep disturbance. Wilcoxon signed-rank tests were conducted to evaluate changes in PROs between timepoints (pre-treatment, on-treatment, post-treatment). Logistic regression was used to compare PROs between CIPN and non-CIPN groups at each time point, after adjustment for age, race, and pre-treatment CIPN score. Results were adjusted for multiple comparisons using a false discovery rate (FDR) procedure (FDR *P* < .05). A random forest model using 19 patient features was employed to build a CIPN predictive model.

**Results:**

Out of 229 patients, 75% developed CIPN. Higher depression scores were associated with CIPN development across the three time periods: pre-treatment (OR = 1.20, FDR *P* = 5.3 × 10^−^3), on-treatment (OR = 1.32, FDR *P* = 4.4 × 10^−^4), and post-treatment (OR = 1.24, FDR *P* = 4.3 × 10^−^3). Similarly, pre-treatment PROMIS physical function T scores were lower among patients who developed CIPN (FDR *P* = 1.30 × 10^−2^), on-treatment (FDR *P* = 5.78 × 10^−6^) and post-treatment (FDR *P* = 8.7 × 10^−5^). On treatment anxiety scores were higher on-treatment in patients experiencing CIPN (OR = 1.30, FDR *P* = 4.3 × 10^−3^). Patients with obesity were 3.78 times more likely to develop CIPN compared to those with normal weight (FDR *P* = 3 × 10^−3^). Patients with CIPN did not report experiencing higher levels of sleep disturbance (FDR *P* > .05). The random forest predictive model reached an accuracy of 84% with body mass index (BMI), physical function score and general anxiety score as the most important predictors.

**Conclusion:**

These findings indicate that those experiencing CIPN were more likely to report reduced physical functioning, increased anxiety and depression, and have higher BMIs. As new multi-modal approaches are developed for CIPN, pre-treatment physical functioning, BMI and other patient reported measures may provide predictive ability.

## Introduction

Chemotherapy-induced peripheral neuropathy (CIPN) is a common side effect that occurs in over 50% of patients receiving taxane chemotherapy (1). Of those, 30%–40% develop CIPN symptoms severe enough that their treatment is delayed, decreased, or discontinued (2–4). Dose modifications put patients at risk for reduced treatment efficacy and survival (5–7). Predictive models that identify patients at high risk for CIPN may allow providers to personalize taxane treatment, manage symptoms, and reduce severity.

Prior work has explored the use of predictive models for CIPN (8); however, none are widely adopted in clinical practice. A potential limitation of previously developed predictive models is the absence of patient-reported outcomes (PROs) collected pre-treatment. Prior work has also indicated that anxiety and possibly depression (9–11), higher body mass index (12, 13), diminished physical function (14), and sleep dysfunction (15) are associated with worsening CIPN.

In this study, we explored relationships between the development of moderate-to-severe CIPN in patients with breast cancer treated with taxanes and selected PROs, including anxiety, depression, weight, physical function, and sleep disturbance.

This study aimed to characterize how PROs change over the course of taxane treatment and to determine whether incorporating PROs into a predictive model using a machine learning approach has potential to predict which patients will develop moderate-to-severe CIPN before the initiation of taxane treatment. This work to could help to inform the utility of PROs in future efforts to develop clinical decision-making tools for the management of patients receiving taxane chemotherapy. We hypothesize that PROs collected before taxane therapy is initiated will improve model predictions for which patients will develop CIPN.

## Methods

### Study design

This was a prospective observational study over 12 months and was reviewed and approved by the Cleveland Clinic and University of Utah Institutional Review Boards (IRB #20-908). Females presenting with breast cancer at the Cleveland Clinic and the University of Utah were screened for eligibility, and eligible patients signed an informed consent.

Inclusion criteria included patients older than 18 years of age, female, diagnosed with breast cancer stages I, II, or III, without evidence of distant metastasis, and prescribed one of the following taxane treatment regimens: taxol—weekly for 12 weeks, taxol—every two weeks for a total of four cycles, taxotere—every three weeks, Abraxane—weekly or every 3 weeks. Taxane combinations with pembrolizumab were permitted.

Exclusion criteria included patients that were (i) unable or unwilling to provide informed consent, (ii) receiving treatment with other adjuvant chemotherapies known to have neuropathy as a side effect including cisplatin, folinic acid, oxaliplatin, fluorouracil, vincristine, vinblastine, vinorelbine, eribulin, ixabepilone, or oral paclitaxel, (iii) with history of opioid use for more than three doses in the last seven days prior to enrollment, (iv) on daily non-steroidal anti-inflammatory drug use for four weeks prior to study enrollment, except for intermittent use of no more than five doses per week for pain and low-dose aspirin of 81 mg daily, (v) on chronic steroid therapy greater than a physiological replacement dose 10 mg daily prednisone for managing inflammatory conditions such as arthritis, asthma, or inflammatory bowel disease, (vi) had febrile illness within two weeks prior to enrollment, (vii) had diagnosis of neuropathic pain syndrome such as diabetic neuropathy, post-herpetic neuralgia, complex regional pain syndrome, prior to study entry, or (viii) had any other diagnosis, whether physical or psychological, or a physical exam finding that, in the opinion of the investigator, precluded participation.

PROs, demographic data, and clinical data were collected at seven time points: pre-treatment (visit 1), on-treatment (visits 2–4, corresponding to 1, 2, and 3 months of treatment with taxanes), and post-treatment (visits 5–7, corresponding to 6, 9, and 12 months since enrollment).

### Patient-reported outcomes

PROs included assessments of CIPN, anxiety, depression, weight, physical function, and sleep disturbance. The twenty-question European Organization for Research and Treatment of Cancer Quality of Life Questionnaire for CIPN (CIPN20) (16) was used to evaluate the presence and severity of CIPN. Each item on the CIPN20 was rated on a 4-point Likert scale. The raw scores were summed and then linearly converted to a 0–100 scale, where higher scores indicate greater severity of symptoms. To meet the criteria for CIPN development, an increase of at least 8 points from visit 1 (pre-treatment) was required. CIPN scores were adjusted by subtracting the visit 1 score from all subsequent visits, and patients were classified as having developed CIPN if their adjusted score increased by 8 or more points over time. The CIPN20 included 9 questions on sensory symptoms (e.g., tingling, numbness, and burning sensations), 8 questions on motor symptoms (e.g., difficulty walking, opening jars, and climbing stairs), and 3 questions on autonomic symptoms (e.g., dizziness upon standing, difficulty controlling bowels, and bladder control issues). Patients with pre-treatment CIPN20 scores above 15 were considered to already have evidence of neuropathy and were excluded from the analysis to ensure that only new onset CIPN was assessed.

Anxiety was measured using the Generalized Anxiety Disorder 7-item scale (GAD-7). The GAD-7 scores were categorized as none (0–4), mild (5–9), moderate (10–14), and severe (15–21) anxiety (17). Depression was measured using the Patient Health Questionnaire- 8 (PHQ8) (18). The PHQ-8 depression scores were categorized as minimal or no depression (0–4), mild depression (5–9), moderate depression (10–14), moderately severe depression (15–19), and severe depression (20–24) (19, 20). Physical function and sleep disturbance were measured using the Patient-Reported Outcomes Measurement Information System (PROMIS) short form 6 and 6a, respectively (21–23). The raw scores were transformed into T-scores using a conversion table that was developed based on a representative sample of adults from a general United States population (24, 25). Physical function T-scores were categorized as normal or high functioning (≥50), mild physical limitations (40–49), moderate physical limitations (30–39), and severe physical limitations (<30) (23, 26). Sleep disturbance T-scores were categorized as normal (≤55), mild sleep disturbance (56–59), moderate sleep disturbance (60–69), and severe sleep disturbance (≥70).

### Demographic and clinical data

Demographic data included age, race, height, weight, body mass index (BMI), ethnicity, and smoking history. Clinical data included tumor staging, tumor metastasis, type of chemotherapy regimen, use of radiation therapy, and the Charlson comorbidity index (CCI). CCI was classified as low, moderate, and high comorbidity for scores of 0–1, 2, and 3 or higher, respectively. BMI was classified as normal (<25 kg/m^2^), overweight (25–29.9 kg/m^2^), and obese (≥30 kg/m^2^).

### Statistical analysis and predictive model construction

All statistical analysis was performed using the R statistical software (v.4.2.2) (27). Demographic and clinical data were presented as medians (interquartile ranges). Patients were organized into two observational groups: CIPN and non-CIPN. The CIPN group was considered to have moderate-to-severe CIPN, defined as an 8-point linearized increase from pre-treatment score. A Wilcoxon signed-rank test was used for paired comparison of PROs at each of the seven observation time points within the CIPN and non-CIPN groups. To account for multiple comparisons, *P* values were adjusted using a false discovery rate (FDR) approach between CIPN and non-CIPN groups (28). An FDR-adjusted *P* < .05 was considered statistically significant.

To assess associations between age, race, BMI, physical function, sleep disturbance, GAD-7, and PHQ-8 depression scores with the likelihood of developing CIPN, seven logistic regression models were built. Each model was adjusted for potential confounding variables, including pre-treatment CIPN scores, age, and race. Odds ratios (ORs) and 95% confidence intervals (CIs) were calculated to quantify the associations. Data processing and visualization were performed using R *dplyr* (29) and *ggplot2* (30) packages, respectively.

A random forest machine learning model was built using the *randomForest* R package (31, 32). The model was constructed using 500 trees and three variables per split, consisting of randomly selected clinical features before taxane therapy was initiated to predict CIPN onset. For each individual decision tree, the model was configured to consider three randomly selected predictor variables at each split. Each split selected predictor variables consisted of randomly selected clinical features before taxane therapy was initiated to predict CIPN onset from 19 features including the GAD-7 score, pain catastrophizing score (PCS), PHQ-8 score, Brief Pain Inventory score (BPI), physical function and sleep disturbance scores, pre-treatment CIPN scores, age, race, ethnicity, smoking status, CCI (33), BMI, tumor and treatment characteristics.

The R package, *ROSE* (34), was used to handle class imbalance. Class imbalance addresses model training challenges when one group is overrepresented compared to the other group. A hybrid method was employed that both undersampled the overrepresented CIPN group and oversampled the underrepresented non-CIPN group. A random forest model was trained on 80% of the data and 20% was used to test its performance. Training model error was estimated using out-of-bag (OOB) error, a built-in validation metric in random forest (31, 35). A lower OOB error indicates higher training model accuracy. Model performance was evaluated by measuring test set accuracy, defined as the proportion of correctly classified patients that developed and did not develop CIPN, model sensitivity, and model specificity. In addition, Brier score was used to evaluate model calibration (36). The data used to construct the model is provided in the Supplementary Material.

## Results

Two hundred and twenty-nine patients enrolled from January 2021 to June 2024 and completed the 12-month study period. One hundred and thirty-five and 94 patients were enrolled at the Cleveland Clinic (CC) and the University of Utah (UU), respectively. The mean age of patients was 53 years (SD = 12), and 82% of patients identified as white, and smoking prevalence was 29%. Stage 1A and 2A were the most prevalent cancer stages, accounting for 22% and 26% of cases, respectively. The majority of patients had moderately differentiated (grade 2, 44%) or poorly differentiated (grade 3, 45%) tumors, with most tumors being localized (T1, 36%, and T2, 44%) with limited or no lymph node involvement (*N*0 = 53%, N1 = 52%). The majority of patients did not receive hormone therapy (86%) or radiation treatment (88%). Thirty-one percent received paclitaxel (Taxol) weekly for 12 weeks, 13% received paclitaxel (Taxol) every 2 weeks for four cycles, 4% received paclitaxel wrapped in albumen (Abaraxane), 38% received docetaxel (Taxotere) every 3 weeks, and 2% received other taxane treatment regimens. Table 1 presents the demographics, clinical characteristics, and treatment details by CIPN group. Additional details regarding the patient's characteristics are presented in the Supplementary Results.

**Table 1:** Demographic and clinical characteristics of patients with breast cancer treated with taxane chemotherapy.

Variable		CIPN group	NON-CIPN group	FDR *P* value
		(*N* = 173)	(*N* = 56)	
Demographic Characteristics				
Age [Mean (SD)]		53 (12)	52 (13)	0.60
BMI [Mean (SD)]		30.49 (7.76)	28.31 (7.15)	0.01
	American Indian or Alaska Native	2	0	0.80
	Asian	3	6	
	Black or African American	6	4	
Race (%)	Native Hawaiian or Pacific Islander	2	2	
	Not Reported	4	2	
	White	83	86	
Ethnic (%)	Hispanic or Latino	6	4	0.80
	Not Hispanic or Latino	89	92	
	Not Reported	5	4	
Smoke (%)	No	69	72	0.80
	Yes	31	28	
Treatment				
Radiation (%)	No	89	92	0.70
	Unknown	3	6	
	Yes	8	2	
Hormone (%)	No	86	88	0.80
	Unknown	10	6	
	Yes	4	6	
Chemo Regimen (%)	Nab-paclitaxel (Abraxane)—weekly or every 3 weeks	4	4	0.80
	Paclitaxel (Taxol)—weekly × 12 weeks	41	30	
	Paclitaxel (Taxol) every 2 weeks 4 times	16	14	
	Doceltaxel (Taxotere)—every 3 weeks	37	50	
	Other Regimens^a^	2	2	
Clinical Characteristics				
Her2 Receptor (%)	HER2−	60	67	0.80
	HER2+	40	32	
	Mixed	1	2	
Estrogen Receptor (%)	ER−	39	24	0.70
	ER+	60	76	
	Mixed	1	0	
Progesterone receptor (%)	Mixed	2	0	0.70
	PR−	48	39	
	PR+	50	61	
Grade (%)	Grade 1	6	12	0.80
	Grade 2	46	41	
	Grade 3	45	45	
	Other	1	2	
	Unknown	3	0	
Tumor status (%)	T1	39	37	0.80
	T2	42	49	
	T3	16	12	
	T4	3	2	
	TX	1	0	
Lymph nodes (%)	N0	52	55	0.7
	N1	39	33	
	N2	6	10	
	N3	3	0	
	NX	0	2	
Metastasis (%)	M0	82	90	0.7
	MX	18	10	

Values are presented as mean (standard deviation) or percentage.

CIPN, chemotherapy-induced peripheral neuropathy; BMI, body mass index; ER, estrogen receptor; PR, progesterone receptor; HER2, human epidermal growth factor receptor 2; FDR, false discovery rate.

a Other regimens include taxane-based combinations with or without pembrolizumab (checkpoint inhibitor), HER2-targeted therapies (e.g., TCHP, AMB TCH-P, Phesgo), and doxorubicin monotherapy.

### Patient reported outcomes

**Figure 1:**
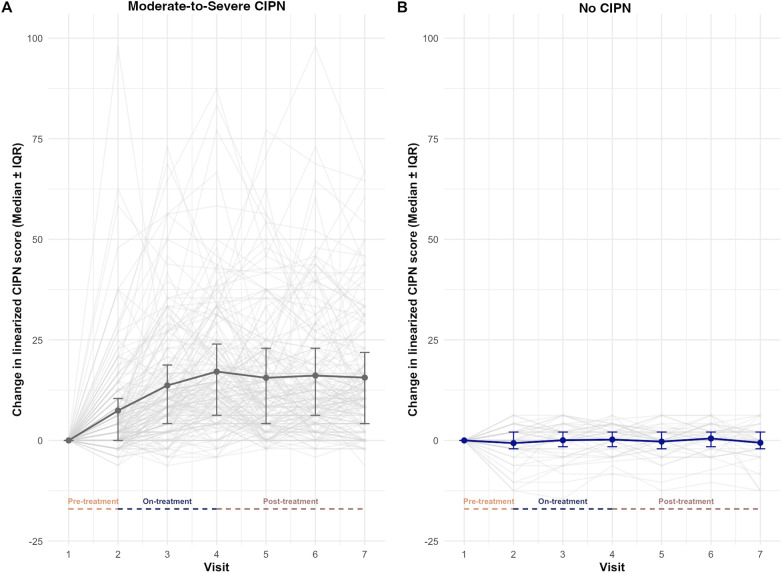
Changes in linearized chemotherapy induced peripheral neuropathy (CIPN) scores over the 12 month observation period from pre-treatment values in patients with and without CIPN. **(A)** Variability of patient CIPN20 scores in patients deemed to have moderate-to-severe CIPN20. Light grey lines represent individual linearized CIPN20 scores reported at each of seven study visits. The dark grey solid line and whiskers represent the median CIPN20 and interquartile range (IQR) CIPN scores, respectively. **(B)** Patients that did not reach the threshold for moderate-to-severe CIPN had lower variability among their scores, as can be seen in the reduced scatter of the light grey lines and smaller interquartile range. The dark solid line and whiskers represent the median linearized CIPN2) scores and IQR, respectively at each of the seven study visits. Pre-treatment, on-treatment, and post-treatment phases of the treatment are annotated at the bottom of each figure.

CIPN scores demonstrated significant inter- and intra-individual variability once treatment started and persisted throughout the 12-month observation period (Figure 1). Although, it varied significantly for each person, patients with moderate-to-severe CIPN had their scores increase progressively during the on-treatment period with median scores rising notably after treatment initiation, and symptoms persisted throughout the post-treatment follow up period (Figure 1A). In contrast, patients without CIPN exhibited minimal change in symptom scores over the same period, with much less variability (Figure 1B). One hundred and seventy-three out of the 229 patients (75%) met the criteria of CIPN. In patients with CIPN, sensory symptoms occurred in 75% of patients with a median score of 8 (IQR: 0–19). Motor symptoms occurred in 27% of patients, with a median of 9 (IQR: 0–18), and autonomic symptoms occurred in 17% of patients, with a median of 17 (IQR: 0–17). Across the entire cohort, the anxiety (GAD-7) scores decreased from 4 (IQR: 1–7) to 2 (IQR 1–5, FDR *P* = 7.46 × 10^−9^) and 2 (IQR: 0–5, FDR *P* = 7.54 × 10^−9^) for pre-, on- and post-treatment scores, respectively. The median pre-treatment (PHQ-8) score was 4 (IQR: 1–7) and decreased to 3 (IQR: 1–6, FDR *P* *=* .03), when all patients were combined (Table 2). The median physical function T-score decreased across the cohort from 48.9 (IQR: 43.2–59) pre-treatment to 45.4 (IQR: 40.4–51.3) on-treatment, followed by an increase to 47.2 (41.13–51.3) post-treatment (Table 3). The median sleep disturbance T-score was stable across three time points, 59.7 (IQR: 56.1–62.3) pre-treatment, 59.76 (IQR: 57.7–61.46) on-treatment, and 59.53 (IQR: 56.65–61) post-treatment (Table 4).

**Table 2:** Chemotherapy induced peripheral neuropathy and patient health questionnaire—8 (PHQ-8) depression scores.

Time-points	PHQ-8 score			
	All patients combined	CIPN	Non-CIPN	CIPN
				vs.
	(*N* = 229)	(*N* = 173)	(*N* = 56)	Non-CIPN
	Median (IQR)	Median (IQR)	Median (IQR)	Odds ratio (FDR *P*-value)
Pre-treatment	4 (1–7)	4 (2–7)	3 (1–5)	1.20 (5.3 × 10^−3^)
On-treatment	4 (1–7)	5 (3–8)	3 (1–6)	1.32 (4.4 × 10^−4^)
Post-treatment	4 (2–6)	4 (2–7)	3 (1–5)	1.24 (4.3 × 10^−3^)

**Table 3:** Chemotherapy induced peripheral neuropathy and patient-reported outcomes measurement information system (PROMIS) physical function T scores.

Time-points	PROMIS physical function T score			
	All patients combined	CIPN	Non-CIPN	CIPN
				vs.
	(*N* = 229)	(*N* = 173)	(*N* = 56)	Non-CIPN
	Median (IQR)	Median (IQR)	Median (IQR)	Odds ratio (FDR *P*-value)
Pre-treatment	49 (43–59)	48 (42–59)	51 (44–59)	0.98 (1.30 × 10^−2^)
On-treatment	45 (40–51)	44 (40–49)	50 (43–56)	0.87 (5.8 × 10^−6^)
Post-treatment	47 (41–51)	46 (41–52)	52 (43–59)	0.89 (8.7 × 10^−5^)

**Table 4:** Chemotherapy induced peripheral neuropathy and patient-reported outcomes measurement information system (PROMIS) sleep disturbance T scores.

Time-points	PROMIS sleep disturbance T score			
	All patients combined	CIPN	Non-CIPN	CIPN
				vs.
	(*N* = 229)	(*N* = 173)	(*N* = 56)	Non-CIPN
	Median (IQR)	Median (IQR)	Median (IQR)	Odds ratio (FDR *P*-value)
Pre-treatment	60 (56–62)	60 (56–62)	60 (56–61)	0.99 (0.87)
On-treatment	60 (58–61)	59 (57–61)	60 (57–61)	0.95 (0.41)
Post-treatment	60 (57–61)	60 (58–62)	60 (58–61)	0.93 (0.30)

### Associations of patient reported outcomes and demographic and clinical data with onset of CIPN

Patients who developed CIPN had a higher mean BMI (Mean = 30.49, SD 7.76) compared to patients who did not develop CIPN (Mean = 28.31, SD 7.15) (OR = 1.086; FDR *P* = 0.01) (Table 1). Among patients with CIPN, 25% were classified as having normal weight, 30% as having overweight, 46% as having obesity. In comparison, 40% of patients who did not develop CIPN were classified as having normal weight, 33% as having overweight, and 27% as having obesity. Patients with obesity, but not overweight, were more likely to develop CIPN compared to patients with normal weight (OR = 3.78, 95% CI 1.58–9.65, FDR *P* = 3 × 10^−3^). There were no statistically significant associations between age (FDR *P* *=* .50) or race and developing CIPN; however, our cohort lacked racial diversity. Tables 2–5 present anxiety, depression, physical function, and sleep disturbance scores by CIPN group, respectively. There was no association between pre-treatment anxiety and developing CIPN (*P* > .05) (Table 5). Anxiety scores decreased in both groups on- and post-taxane treatment, but they were significantly higher in the CIPN group on-treatment (OR = 1.3, FDR *p* = 4.3 × 10^−3^) and post-treatment (OR = 1.15, FDR *P* =  0.06) (Table 5). The CIPN group had higher depression scores pre-treatment (OR = 1.20, 5.3 × 10^−3^), on-treatment (OR =  1.32, 4.4 × 10^−4^), and post-treatment (OR =  1.24, 4.3 × 10^−3^) compared to the non-CIPN group (Table 2). Physical function scores were higher in the non-CIPN group pre-treatment (Median = 51) and remained consistent while on-treatment (Median = 50) and post-treatment (Median = 52) (FDR *P* > .05) (Table 3). However, the CIPN group was at a lower level of physical functioning pre-treatment (Median=48) compared to the non-CIPN group (Median = 51), and dropped significantly on-treatment (Median = 44, FDR *P* = 1.42 × 10^−11^), and failed to fully recover post-treatment (Median = 46, FDR *P* = 1.2 × 10^−3^) (Table 3).

**Table 5:** Chemotherapy induced peripheral neuropathy and generalized anxiety disorder—7 (GAD-7) scores.

Time-points	GAD-7 score			
	All patients combined	CIPN	Non-CIPN	CIPN
				vs.
	(*N* = 229)	(*N* = 173)	(*N* = 56)	Non-CIPN
	Median (IQR)	Median (IQR)	Median (IQR)	Odds ratio (FDR *P*-value)
Pre-treatment	4 (1–7)	4 (1–7)	4 (1–7)	1.04 (0.4)
On-treatment	2 (1–5)	3 (1–6)	1 (0–3)	1.3 (4.3 × 10^−3^)
Post-treatment	2 (0–5)	2 (1–5)	1 (0–4)	1.15 (6.1 × 10^−2^)

There were no differences in sleep disturbance between CIPN and non-CIPN groups for pre-, on, and post-treatment (Table 4). No associations were observed between pre-treatment sleep disturbance and likelihood of developing CIPN (OR = 0.99, FDR *P* = .87). Additionally, no significant differences in sleep disturbance were observed at any time point within either the CIPN or non-CIPN groups, nor between the groups on-treatment (OR = 0.95, FDR *P* = .41), or post-treatment (OR = 0.93, FDR *P* = .30) (Table 4).

### Machine-learning model for prediction of CIPN

**Figure 2:**
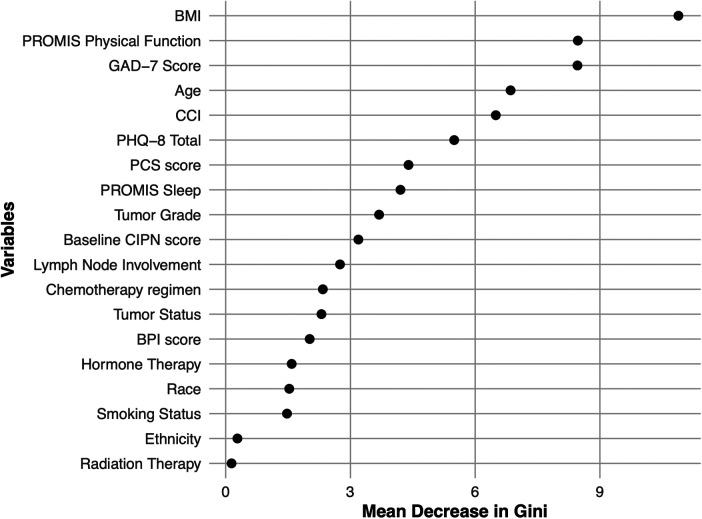
Model feature importance for predictions of moderate to severe chemotherapy induced peripheral neuropathy (CIPN). Features included in the random forest model are shown on the *y*-axis. The *x*-axis shows the mean decrease in Gini score for each variable, which represents a measure of feature importance for predictions, with higher values indicating a greater contribution of that feature to the model predictions. GAD-7, generalized anxiety disorder-7 score; PCS, pain catastrophizing scale score; PHQ-8, patient health questionnaire-8 (PHQ-8) depression score; BPI, brief pain inventory score, PROMIS, patient-reported outcomes measurement information system physical function and sleep disturbance scores; BMI, body mass index; CCI, Charlson comorbidity index.

A random forest model built from 19 pre-treatment clinical features to predict moderate-to-severe CIPN. The training model's OOB was 4.2%, and the model predicted CIPN with 84% accuracy in the withheld test set. Model sensitivity and specificity were 85% and 76%, respectively. Among the 19 features used to build this model, BMI, PROMIS physical function, GAD-7 scores, age, CCI, and PHQ-8 were the most important predictors (Figure 2). The Brier Score was 0.176 indicating good calibration.

## Discussion

This longitudinal study of clinical data and patient-reported outcomes in 229 patients with breast cancer provided observations of CIPN trajectories and patient reported outcomes pre-treatment, on-treatment, and post-treatment with taxanes. Patients enrolled in this study were of similar age, tumor staging, tumor subtypes, and taxane treatment regimens compared to prior published work (2–4).

The CIPN scores over time were similar to what has been previously reported (37). CIPN scores were variable, especially in the CIPN group, suggesting the causes of CIPN may be multifactorial, making it difficult to predict and manage (Figure 1). Consistent with the understanding of CIPN mechanisms, the sensory symptoms of the CIPN score were more prevalent than motor or autonomic symptoms, suggesting that sensory nerves may be more at risk for damage than other nerve fiber types (38).

Anxiety scores indicated patients were minimally anxious pre-, on-, and post-treatment. The lack of anxiety suggests this measure is unlikely to be useful in predicting the onset of CIPN. This result is in contrast to findings by several investigators who report a positive association between anxiety and the development of CIPN (10, 39) and established a 30% increase in risk of developing CIPN for every unit increase in the GAD-7 score.

The average depression scores in our study cohort, for the most part, suggested minimal to no depression. In the CIPN group, the average scores on-treatment were high enough to suggest mild depression. Our results suggest that a one unit increase in the depression score produces a 20% higher likelihood of developing CIPN (OR = 1.20; FDR *P* = 5.3 × 10^−3^) (Table 2). These findings are consistent with those of Lee et al., who studied patients in South Korea, that either received taxane-containing or non-taxane chemotherapy and demonstrated a strong correlation between increased depression levels and CIPN severity (39). Notably, Bennedsgaard et al. also found that patients treated with taxanes experienced greater psychological distress as demonstrated through depressive symptoms compared to other conventional breast cancer treatments (40). Consistent with prior work (41), our findings suggest that CIPN may be more closely linked to feelings of depression than anxiety, highlighting a potentially important distinction in the psychological burden of chemotherapy. A better understanding of how psychological factors contribute to the onset and severity of CIPN is needed to drive meaningful improvements in clinical management, especially in light of the observed differences between anxiety and depression symptoms. Duloxetine, a serotonin and norepinephrine reuptake inhibitor (SSNRI), is used to treat major depressive disorder, generalized anxiety disorder, diabetic neuropathy, and is used off-label for the treatment of CIPN, indicating an important link between these conditions (42). Studies are needed to determine whether early psychological assessment and management could improve the mental health of these patients and potentially alleviate some of the impacts on patient quality of life, as well as the symptoms of CIPN. In summary, our findings, along with those of other studies, suggest that depression symptoms existing prior to the onset of chemotherapy demonstrate an increased risk of CIPN development.

We observed a small difference in physical function between those patients who developed CIPN and those who did not. Prior to treatment, those who did not develop CIPN were more likely to report normal or high functioning physical function (T-scores ≥ 50), whereas patients who did develop CIPN were more likely to report mild physical limitations (T-scores 40–49). A T-score change of 2–6 points is considered to be the threshold for a minimal clinically important difference (43). Our data are consistent with the results of a recent study of pancreatic cancer patients undergoing taxane-based therapy, which found that pre-treatment physical function, specifically mobility and balance, predicted the severity of CIPN six months post-treatment (44). The importance of physical pre-treatment factors and their ability to modify the risk and severity of CIPN warrants further investigation. Both groups displayed a modest decrease in physical functioning on- and post-treatment, but for the most part, remained in the normal or mild physical limitation range. Our data showed that every increased unit for pre-treatment physical function T-scores was associated with a 2% decreased likelihood of developing CIPN (OR = 0.98, FDR *P* = 1.30 × 10^−2^) (Table 3). Post-treatment, both groups reported improvement in physical function, but the CIPN group failed to reach its pre-treatment levels, whereas the non-CIPN group demonstrated a complete recovery of physical functioning (Table 3). These results suggest that physical function assessments may be useful, as part of a broader panel of predictors, for predicting which patients develop CIPN.

Our results suggest that obesity is a predictor of patients at risk of developing CIPN, and this is consistent with prior work. For example, data from the Pathways Study identified a strong association between BMI and CIPN risk (45). Specifically, the Pathways Study reported increased odds of CIPN in patients with overweight (OR = 2.37, 95% CI 1.19–4.88) and obesity (OR = 3.21, 95% CI 1.52–7.02) compared to normal weight patients at 24 months post-treatment (45). In our study, we observed a similar effect size for the association between obesity and CIPN, but did not observe a significant association between overweight and CIPN (Table 1). Obesity has been shown to increase the risk of peripheral neuropathy, even in the absence of hyperglycemia (30, 31). The mechanism by which obesity may lead to increased risk of peripheral neuropathy is poorly understood. However, some mechanisms have been proposed, such as modified expression of toll-like receptors in patients with obesity, that may result in neuropathic symptoms (46).

Our study identified depression, obesity, and physical function as potentially modifiable risk factors in patients with CIPN. A randomized controlled trial previously demonstrated that patients that underwent strength training and balancing exercise experienced reduced CIPN pain and increased quality of life scores, supporting that interventions that improve physical health may serve as viable interventions for improving CIPN occurrence and severity (47). Furthermore, studies have reported that organized exercise and diet counseling during chemotherapy led to significant reductions in BMI, improvements in physical functioning, and reductions in fatigue, anxiety, and depression (48, 49). This is also supported by the American Society for Clinical Oncology, which recommends exercise and weight management interventions to promote physical and psychological well-being (50).

While evidence for long-term prevention of CIPN remains in development, these findings suggest that early detection of at-risk patients could allow for early referral to evidence-based therapy, including behavioral therapy, structured exercise, and weight reduction programs. This warrants further investigation to determine if a pre-treatment assessment representing mental health, physical functioning, and obesity could sufficiently identify patients for targeted preventive care interventions to improve patient outcomes. Predicting which patients face the highest risk of CIPN could allow for more efficient allocation of resources toward targeted interventions.

We developed a predictive model using a random forest algorithm to investigate whether pre-treatment clinical and psychological characteristics could be used to identify patients at risk of developing moderate-to-severe CIPN. The model was built from 19 pre-treatment clinical features and achieved good predictive performance, with 84% accuracy, with high sensitivity and specificity of 85% and 76%, respectively. This level of accuracy is very promising, indicating potential to identify patients at high risk of CIPN for early intervention. Among the 19 features used to build this model, BMI, PROMIS physical function, GAD-7, age, CCI and PHQ-8 scores had the highest mean decrease in Gini score, indicating a greater contribution to the model's prediction accuracy (Figure 2). It is important to note that Gini score does not specifically indicate the predictive ability of the individual feature, it represents a relative contribution of each feature for the overall model predictive performance. Future studies are warranted to identify other predictors, such as biomarkers (e.g., mRNA expression, single-nucleotide variants, metabolites) to improve model predictions.

There are several important limitations that should be considered when interpreting these results. The study population might not be representative of all the patients with breast cancer, and the sample size did not allow for consideration of all potential variations in demographics, cancer stages, and treatment protocols across different geographic areas or healthcare systems. PROs provide valuable insight, yet they may introduce bias (e.g., recall bias, social desirability bias), potentially affecting the accuracy of the study outcomes. Although we used random forest as our machine learning approach, which is an established methodology recognized for its robust performance (31), additional work is needed in independent patient cohorts to establish real-world model performance. We opted to use random forest for our modeling approach due to our cohort size and the relative robustness to model overfitting with random forest; however, with larger cohorts, other methodologies, such as support vector machines or neural networks could be evaluated to determine if greater predictivity could be achieved. Because our cohort was not representative of all patients with breast cancer, the proposed model should be evaluated in larger more diverse cohorts, where model performance can be assessed and accuracy can be evaluated in all types of patients to establish the appropriate context of clinical use.

In summary, our study highlights important relationships between CIPN and depression and body habitus in patients with breast cancer undergoing taxane-based chemotherapy. These results support the inclusion of these data into future efforts to build a model that will reliably predict which patients would develop CIPN before taxane treatment was started. The addition of biomarkers may improve the model's ability to predict which patients will develop CIPN. With additional validation, this model could be used to enrich clinical trial populations for emerging CIPN interventions, or to identify individuals that may benefit from evidence-based interventions to improve the quality of life for patients and reduce the likelihood of treatment discontinuation. Future research should focus on understanding the underlying mechanisms that link psychological stress with neuropathy and explore possible biomarkers associated with these mechanisms to improve model predictions and identify effective interventions for CIPN.

## Data Availability

The original contributions presented in the study are included in the article/Supplementary Material, further inquiries can be directed to the corresponding author.
