# Supplementary material for: Time-course based assessment of patient factors and their relationship to chemotherapy induced peripheral neuropathy
Source: Front Pain Res (Lausanne). 2026 Jan 30;7:1619858. doi: 10.3389/fpain.2026.1619858 (PMC12901485; doi:10.3389/fpain.2026.1619858)
Supplement: Supplementary file 1 [file Table1.docx]

**SUPPLEMENTARY MATERIAL**

**Time-course based assessment of patient factors and their relationship to chemotherapy induced peripheral neuropathy**

Carla Bou Dargham, MD^1^, Ken B. Johnson, MD^2^, Alper Sen, MD^2^, Bihua Bie, MD^3^, Emily E. Rhoades, PhD^4^, Jacob Steenblik DNP, MPH, MHA^2^, Courtney Hershberger, PhD^1^, Mei Wei, MD^5^, N. Lynn Henry, MD^6^, Anukriti Sharma, PhD^1^, G. Thomas Budd, MD^3,7,8^, Joseph Foss, MD^3^, Daniel M. Rotroff, PhD^1,7,8,9^

1. Department of Quantitative Health Sciences, Cleveland Clinic Research, Cleveland Clinic, OH, USA
2. Department of Anesthesiology, University of Utah, UT, USA
3. Department of Anesthesiology, Cleveland Clinic, OH, USA
4. Taussig Cancer Institute, Cleveland Clinic, OH, USA
5. Huntsman Cancer Institute, University of Utah, Salt Lake City, UT
6. University of Michigan Medical School, Ann Arbor, MI 48109, USA
7. Cleveland Clinic Lerner College of Medicine, Case Western Reserve University, Cleveland, OH, USA
8. Case Comprehensive Cancer Center, Case Western Reserve University School of Medicine, Cleveland, OH, USA
9. Center for Quantitative Metabolic Research, Cleveland Clinic, Cleveland, OH, USA

**# Corresponding Author**

Daniel M Rotroff, PhD, MSPH

Department of Quantitative Health Sciences,

Cleveland Clinic Research,

Cleveland Clinic,

9500 Euclid Avenue, JJN3-01,

Cleveland, OH 44195, United States

[rotrofd@ccf.org](mailto:rotrofd@ccf.org)

**Supplementary Results**

The mean average age of patients enrolled at CC was slightly higher than at UU, with mean ages of 55 and 51, respectively (FDR *P*=.03). There were no statistically significant differences in ethnicity between sites (FDR *P* >.05), but there were significant differences in self-reported race (FDR *P*=.012). At CC, 79 % of patients identified as white compared to 87% at UU. Smoking prevalence was also higher in CC patients than UU patients with 37% and 19%, respectively (FDR *P*=.015). There were also notable differences between tumor staging across CC and UU sites: Patients enrolled at CC had more advanced disease than patients at UU. This is reflected in the tumor staging (FDR *P*=.012) and in the high proportion of patients with metastases with 32% compared to 7% at CC and UU, respectively (FDR *P*=1x10^-5^). There were also differences in chemotherapy regimens received by patients (FDR *P*=.004). At CC, Taxotere - every 3 weeks was the most common treatment (44%), whereas Taxol - weekly x 12 weeks (52%) was the most common at UU. A higher proportion received Taxol every 2 weeks 4 times at CC (19%) in contrast to 13% at UU. Radiation therapy was also more commonly administered at CC (91%) compared to UU (85%) (FDR *P*=.021).

**Supplementary Tables**

**Table 2: Demographic and clinical characteristics of breast cancer patients treated with taxane chemotherapy**

| **Variable** | | **CC**  **(N = 135)** | **UU**  **(N = 94)** | **FDR *P* value** |
| --- | --- | --- | --- | --- |
| Age (Mean (SD) |  | 55(12.4) | 51 (11.5) | 0.034 |
| **Demographic Characteristics** | | |  |  |
|  | American Indian or Alaska Native | 0.74 | 2.13 | 0.012 |
|  | Asian | 5.93 | 3.19 |  |
|  | Black or African American | 10.37 | 0.00 |  |
| Race (%) | Native Hawaiian or Pacific Islander | 0.74 | 3.19 |  |
|  | Not Reported | 3.70 | 4.26 |  |
|  | White | 78.52 | 87.23 |  |
| Ethnic (%) | Hispanic or Latino | 6.02 | 4.26 | 0.641 |
|  | Not Hispanic or Latino | 87.97 | 90.43 |  |
|  | Not Reported | 6.02 | 5.32 |  |
| Smoke (%) | No | 63.16 | 80.85 | 0.015 |
|  | Yes | 36.84 | 19.15 |  |
| **Treatment** | | |  |  |
| Radiation (%) | No | 91.11 | 85.11 | 0.021 |
|  | Unknown | 5.93 | 2.13 |  |
|  | Yes | 2.96 | 12.77 |  |
| Hormone (%) | No | 89.63 | 81.91 | 0.066 |
|  | Unknown | 8.89 | 9.57 |  |
|  | Yes | 1.48 | 8.51 |  |
| Chemo Regimen (%) | Abraxane - weekly or every 3 weeks | 0.75 | 6.38 | 0.004 |
|  | Other - specify | 3.73 | 0.00 |  |
|  | Taxol - weekly x 12 weeks | 32.84 | 52.13 |  |
|  | Taxol every 2 weeks 4 times | 18.66 | 12.77 |  |
|  | Taxotere - every 3 weeks | 44.03 | 28.72 |  |
| **Clinical Characteristics** |  |  |  |  |
| Her2 Receptor (%) | HER2- | 65.19 | 55.32 | 0.190 |
|  | HER2+ | 33.33 | 44.68 |  |
|  | Mixed | 1.48 | 0.00 |  |
| Estrogen Receptor (%) | ER- | 34.07 | 38.30 | 0.641 |
|  | ER+ | 64.44 | 61.70 |  |
|  | Mixed | 1.48 | 0.00 |  |
| Progesterone receptor (%) | Mixed | 1.48 | 1.06 | 0.906 |
|  | PR- | 45.19 | 50.00 |  |
|  | PR+ | 53.33 | 48.94 |  |
| Grade (%) | Grade 1 | 8.15 | 6.38 | 0.973 |
|  | Grade 2 | 42.96 | 45.74 |  |
|  | Grade 3 | 45.19 | 45.74 |  |
|  | Other | 0.74 | 0.00 |  |
|  | Unknown | 2.96 | 2.13 |  |
| Tumor status (%) | T1 | 45.19 | 23.40 | 0.012 |
|  | T2 | 38.52 | 53.19 |  |
|  | T3 | 11.85 | 21.28 |  |
|  | T4 | 3.70 | 2.13 |  |
|  | TX | 0.74 | 0.00 |  |
| Lymph nodes (%) | N0 | 56.30 | 48.94 | 0.452 |
|  | N1 | 34.81 | 40.43 |  |
|  | N2 | 7.41 | 6.38 |  |
|  | N3 | 0.74 | 4.26 |  |
|  | NX | 0.74 | 0.00 |  |
| Metastasis (%) | M0 | 93.33 | 68.09 | 1 x 10^-5^ |
|  | MX | 6.67 | 31.91 |  |

**Supplementary Figures**


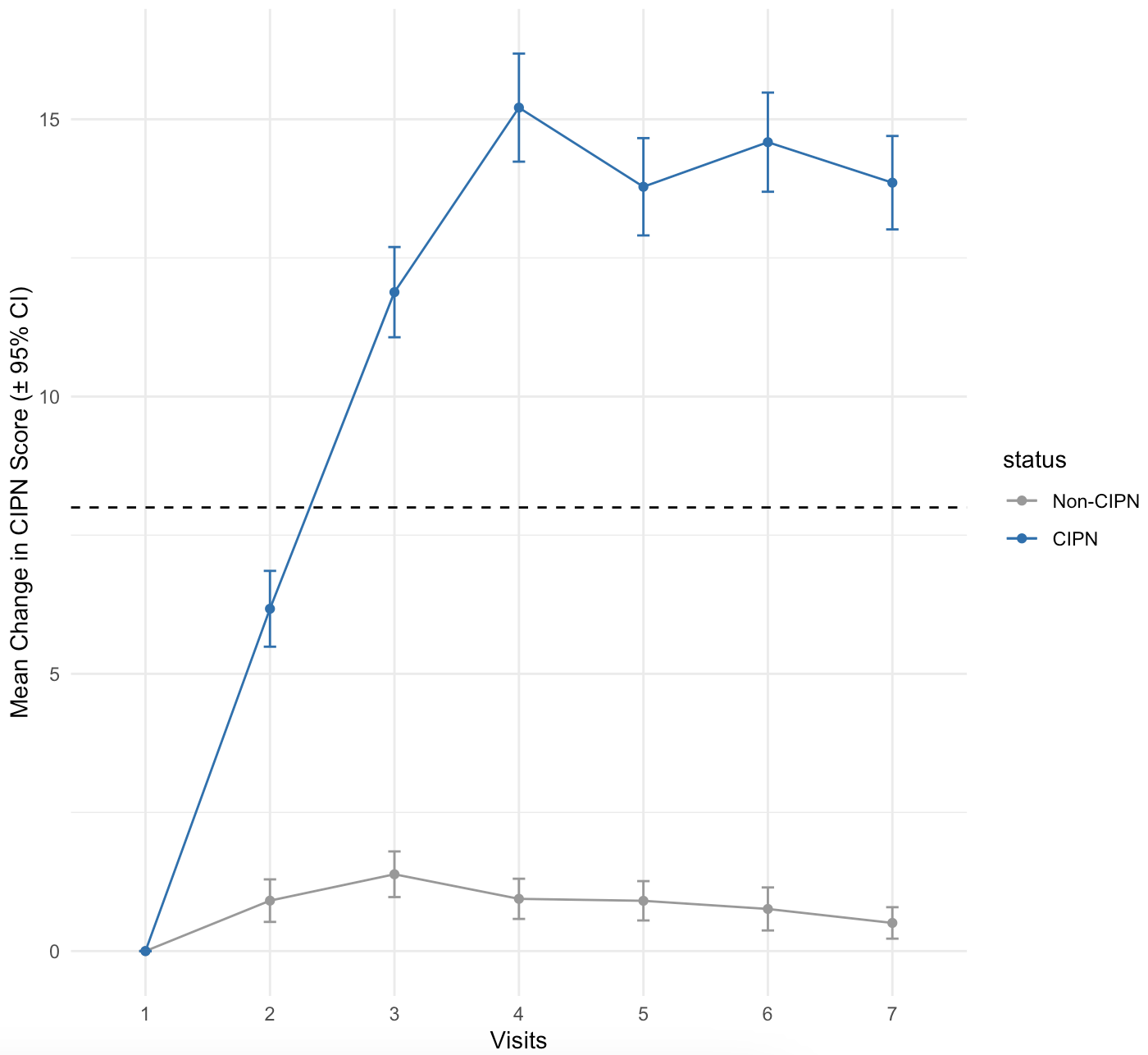


**Figure 1: Line plot displaying the progression of the mean change in CIPN** (Chemotherapy-Induced Peripheral Neuropathy) scores across three phases: before treatment (visit 1), during treatment (visits 2–4), and after treatment (visits 5–7). The plot highlights a clear increase in CIPN symptoms during and after treatment for patients who developed CIPN. The blue line represents participants who developed CIPN, while the gray line represents those who did not develop CIPN. Error bars indicate the 95% confidence intervals around the mean change in CIPN scores from each participant's pre-treatment scores.
